# Differential protease content of mast cells and the processing of IL-33 in *Alternaria alternata* induced allergic airway inflammation in mice

**DOI:** 10.3389/fimmu.2023.1040493

**Published:** 2023-04-19

**Authors:** Olga Krysko, Darya Korsakova, Andrea Teufelberger, Amse De Meyer, Jill Steels, Natalie De Ruyck, Judith van Ovost, Sharon Van Nevel, Gabriele Holtappels, Frauke Coppieters, Mikhail Ivanchenko, Harald Braun, Maria Vedunova, Dmitri V. Krysko, Claus Bachert

**Affiliations:** ^1^Upper Airways Research Laboratory, Department of Head and Skin, Ghent University, Ghent, Belgium; ^2^Institute of Biology and Biomedicine, National Research Lobachevsky State University of Nizhny Novgorod, Nizhny Novgorod, Russia; ^3^Department of Dermatology and Venereology, Medical University of Graz, Graz, Austria; ^4^Center for Medical Genetics Ghent (CMGG), Department of Biomolecular Medicine, Ghent University, Ghent, Belgium; ^5^Institute of Information Technology, Mathematics and Mechanics, National Research Lobachevsky State University of Nizhny Novgorod, Nizhny Novgorod, Russia; ^6^Unit for Structural Biology, VIB-UGent Center for Inflammation Research, Ghent University, Ghent, Belgium; ^7^Unit for Structural Biology, Department of Biochemistry and Microbiology, Ghent University, Ghent, Belgium; ^8^Cell Death Investigation and Therapy Laboratory, Department of Human Structure and Repair, Ghent University, Ghent, Belgium; ^9^Department of Pathophysiology, Sechenov First Moscow State Medical University (Sechenov University), Moscow, Russia; ^10^Department of Otorhinolaryngology - Head and Neck Surgery, University Hospital of Münster, Münster, Germany; ^11^First Affiliated Hospital, Sun Yat-Sen University, International Airway Research Center, Guangzhou, China

**Keywords:** IL-33, mast cells, allergy, type 2 inflammation, protease

## Abstract

**Background:**

Recent *in vitro* studies strongly implicated mast cell-derived proteases as regulators of IL-33 activity by enzymatic cleavage in its central domain. A better understanding of the role of mast cell proteases on IL-33 activity *in vivo* is needed. We aimed to compare the expression of mast cell proteases in C57BL/6 and BALB/c mice, their role in the cleavage of IL-33 cytokine, and their contribution to allergic airway inflammation.

**Results:**

*In vitro*, full-length IL-33 protein was efficiently degraded by mast cell supernatants of BALB/c mice in contrast to the mast cell supernatants from C57BL/6 mice. RNAseq analysis indicated major differences in the gene expression profiles of bone marrow-derived mast cells from C57BL/6 and BALB/c mice. In *Alternaria alternata (Alt)* - treated C57BL/6 mice the full-length form of IL-33 was mainly present, while in BALB/c mice, the processed shorter form of IL-33 was more prominent. The observed cleavage pattern of IL-33 was associated with a nearly complete lack of mast cells and their proteases in the lungs of C57BL/6 mice. While most inflammatory cells were similarly increased in *Alt*-treated C57BL/6 and BALB/c mice, C57BL/6 mice had significantly more eosinophils in the bronchoalveolar lavage fluid and IL-5 protein levels in their lungs than BALB/c mice.

**Conclusion:**

Our study demonstrates that lung mast cells differ in number and protease content between the two tested mouse strains and could affect the processing of IL-33 and inflammatory outcome of *Alt* -induced airway inflammation. We suggest that mast cells and their proteases play a regulatory role in IL-33-induced lung inflammation by limiting its proinflammatory effect *via* the IL-33/ST2 signaling pathway.

## Introduction

Asthma is a heterogeneous inflammatory airway disease with different underlying pathophysiologic mechanisms and disease endotypes, which often manifest by similar clinical complaints (1, 2). In mild asthma, mostly the type 2 phenotype, an eosinophilic immune response is observed, while only a minor percentage of patients show a non-type 2 phenotype (3). In severe asthma, a mixed inflammatory phenotype is also characterized by increased IL-33 levels. Of interest, in children, severe asthma is more common in the case of sensitization to the fungus *Alternaria alternata (Alt)* and is strongly associated with increased IL-33 sputum levels (4). In this regard, a clinical trial has shown that IL-33 targeting could efficiently reduce exacerbations in patients with severe asthma (5). In contrast, patients with mild or moderate asthma have less clinical benefit from an IL-33 targeting therapy. IL-33 is an alarmin, which in allergic airway inflammation is mainly secreted by epithelial cells in response to aeroallergens and signals through a cell surface receptor complex of ST2 (IL-1 receptor-like 1, IL1RL1) and IL-1 receptor accessory protein (IL1RAcP) to stimulate cytokine production in type 2 ILCs and T helper 2 cell, inducing the production of IL-4, IL-5, and IL-13 (6–8). Endogenous serine proteases and cysteine proteases, such as calpains, were suggested to regulate the activity of IL-33 *via* enzymatic cleavage (9–11). Recently, we have demonstrated the contribution of neutrophilic proteases in IL-33 processing in an allergic asthma mouse model using *Alt* (12). IL-33 is produced as a full-length protein lacking a signal sequence by a not entirely clear release mechanism. The cleavage of full-length IL-33 (IL-33_FL_) by human mast cell tryptase, chymase, and cathepsin G *in vitro* results in cleaved IL-33 (IL-33_C_), which is up to 30-fold more potent towards innate lymphoid cells type 2 (ILC2s) (9). It is known that the functional, inflammatory parameters in severe asthma are linked to the presence of activated mast cells (13–15) which, *via* their release of cytokines, proteases, and alarmins, could potentially contribute to the persistence and the exacerbation of the airway inflammation (13, 16, 17); and possibly to the loss of corticosteroid sensitivity in severe asthma (18). In asthmatic patients, bronchoalveolar lavage fluid (BALF) contains increased levels of tryptase (19, 20). The presence of an active form of IL-33 acting on ST2-expressing immune cells could contribute to the sustained type 2 immune response in the airway epithelium (8, 21). Remarkably, in humans, the phenotype of mast cells seems to be different in the same disease with different inflammatory profiles: In mild asthma, tryptase-positive mast cells localize in the submucosa while in severe asthma, mast cells are mostly localized in the airway submucosa and epithelium and express chymase (13, 20). We showed earlier that different inbred mouse strains respond differently to an IL-33 mediated asthma model, using the bacterial allergen, *S. aureus* protease-like protein D (22).

In the current study, we analysed the link between the type of inflammatory response towards *Alt* and the characteristics of mast cells in the two inbred mouse strains C57BL/6 and BALB/c. The strain-dependent mast cell heterogeneity in the regulation of IL-33 processing *in vivo* was addressed.

## Results

### Differential inflammatory response of BALB/c and C57BL/6 mice towards Alt

**Figure 1:**
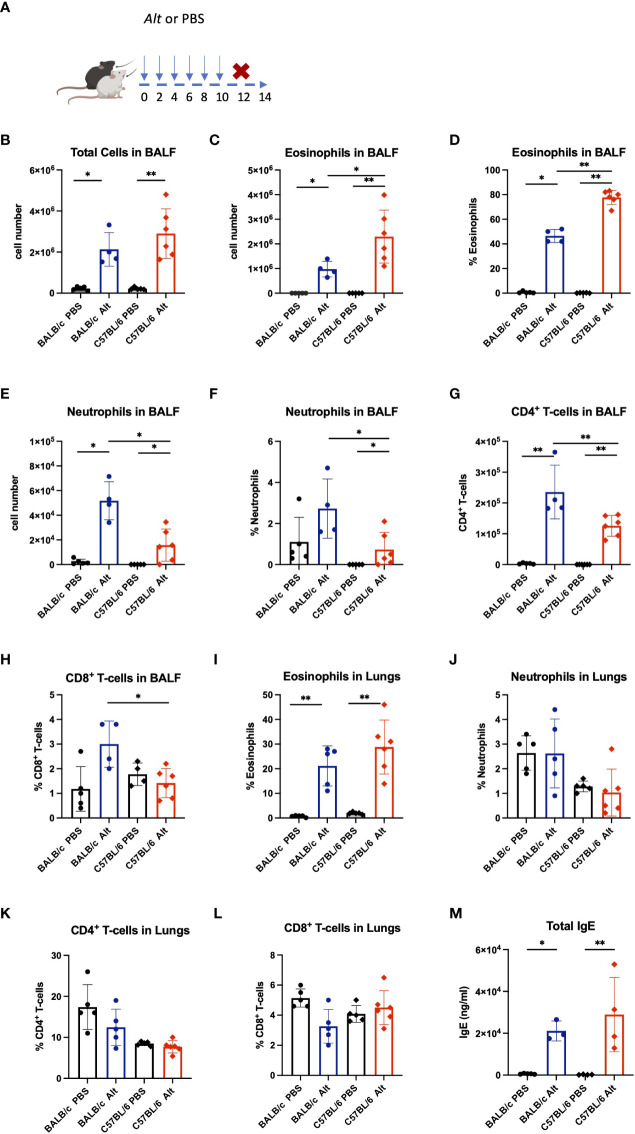
BALB/c and C57BL/6 mice were given six intratracheal applications of 20 *µ*g *Alternaria alternata* (*Alt)* extract in PBS, controls received PBS alone. Forty-eight hours after the last application mice were sacrificed, and experimental samples were collected **(A)**. The absolute cell numbers in the bronchial lavage fluid (BALF) are presented for total cell counts **(B)**, eosinophils **(C)** and neutrophils **(E)** in BALF. The percentage of eosinophils **(D)** and neutrophils **(F)** in BALF are presented. The BALF and lungs were analysed by flow cytometry and the infiltration of eosinophils **(I)**, neutrophils **(J)**, CD4^+^ T cells **(G, K)** and CD8^+^ T cells **(H, L)** in the BALF and lungs are presented as percentage. Mouse serum was collected 48h following the final *Alt* application and the levels of total IgE in serum were determined using enzyme-linked immunosorbent assay **(M)**. The data are presented as scatter plots ± standard deviation (S.D.). Statistical significance between the groups was determined by one-way ANOVA with Dunn’s test for multiple comparisons. N=4-6 per group. *p < 0.05, **p < 0.01.

The IL-33-dependent model of *Alt*-induced airway inflammation was chosen to evaluate an impact on the processing of IL-33 and inflammatory parameters in two commonly used mouse strains (6, 14, 23). C57BL/6 and BALB/c mice received six intratracheal (i.t.) applications of 20 μg *Alt* extract every 48 hours (**Figure 1A**), in a slightly modified application schedule from our previous work (14). The *Alt-*treated BALB/c and C57BL/6 mice showed higher numbers of total BALF cells compared to PBS-treated mice (**Figure 1B**). The BALF of *Alt*-treated C57BL/6 mice contained more eosinophils in BALF as compared to *Alt*-treated BALB/c mice (**Figures 1C, D**). In turn, BALB/c mice had higher neutrophil numbers in the BALF (**Figures 1E, F**). The percentage of CD4^+^ (**Figure 1G**) cells was stronger increased in BALF of BALB/c mice compared to C57BL/6 mice. CD8^+^ cells were higher in BALF of BALB/c mice (**Figure 1H**). Furthermore, in the lungs of both mouse strains, marked eosinophilia was observed (**Figure 1I**), while the percentage of neutrophils remained comparable between *Alt-*treated mice and controls of both mouse strains (**Figure 1J**). Of note, no major difference in the percentages of lung CD4^+^ and CD8^+^ cells was seen (**Figures 1K, L**). Serum IgE levels were higher in *Alt*-treated mice as compared to PBS -treated controls (**Figure 1M**).

### Processing of IL-33 in *Alt*-induced allergic airway inflammation in C57BL/6 and BALB/c mice

**Figure 2:**
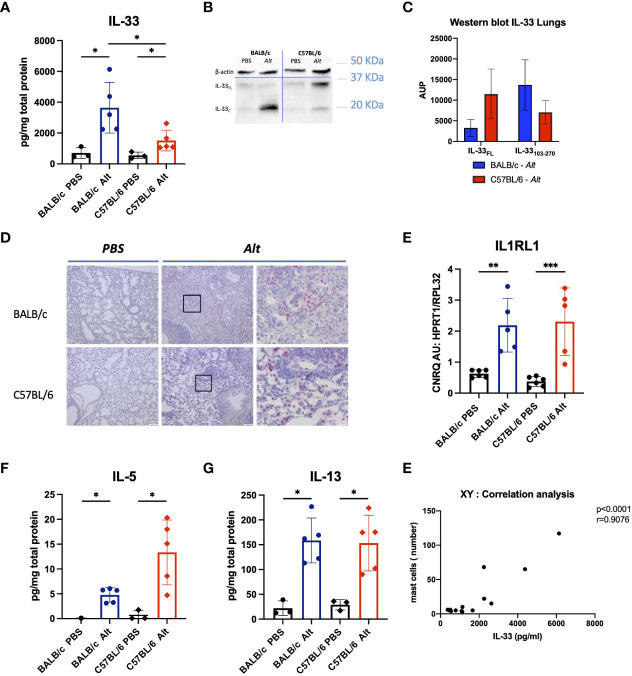
The analysis of cytokine response in the lungs of mice with intratracheal applications of 20 *µ*g of *Alt* extract and PBS-treated controls. The levels of IL-33 **(A)** in the lung homogenates were measured by Luminex. Full-length IL-33 (IL-33_FL_) and cleaved forms (IL-33_C_) were analysed by western blotting and one representative blot is shown **(B)** and area under the peak (AUP) was quantified using ImageJ software **(C)**. Representative images of IL-33 immunostaining in the lungs of C57BL/6 and BALB/c treated with PBS or the *Alt* extract for six times every 48 hours **(D)**. *IL1rl1* gene expression levels analysed in lungs by RT-qPCR **(E)**. The levels of IL-5 **(F)**, IL-13 **(G)** in the lung homogenates were measured by Luminex. The data are presented as scatter plot with a bar ± standard deviation (S.D). Statistical significance between the groups was determined by one-way ANOVA with Dunn’s test for multiple comparisons. N=4-6 per group. *p < 0.05, **p < 0.01, ***p < 0.001.

Notably, despite the comparable degree of inflammatory response in the lungs of C57BL/6 and BALB/c mice, IL-33 levels were significantly higher in BALB/c than in C57BL/6 (**Figure 2A**). The expression of a cleaved/mature form of IL-33 (IL-33_C_; ~ 18 kDa) was higher in the lungs of BALB/c mice, while in C57BL/6 mice, the non-processed full-length form of IL-33 (IL-33_FL_ ~ 30 kDa) was more abundant (**Figures 2B, C**). IL-33 positive cells were abundantly present in the lungs of mice after *Alt* treatment (**Figure 2D**). RT-PCR didn’t show differences between both strains in the respective treatment groups in the expression of IL-33 receptor *IL1Rl1* (**Figure 2E**). Interestingly, IL-5 and IL-13 levels tend to increase in both mouse strains treated with *Alt* (**Figures 2F, G**), while IL-4 and G-CSF were slightly but not significantly increased in both mouse strains, and IL-25 levels in the lungs were comparable in all experimental groups tested (**Figure S1**).

### Increased mast cell numbers and mast cell protease expression in the lungs of *Alt*-treated BALB/c mice

**Figure 3:**
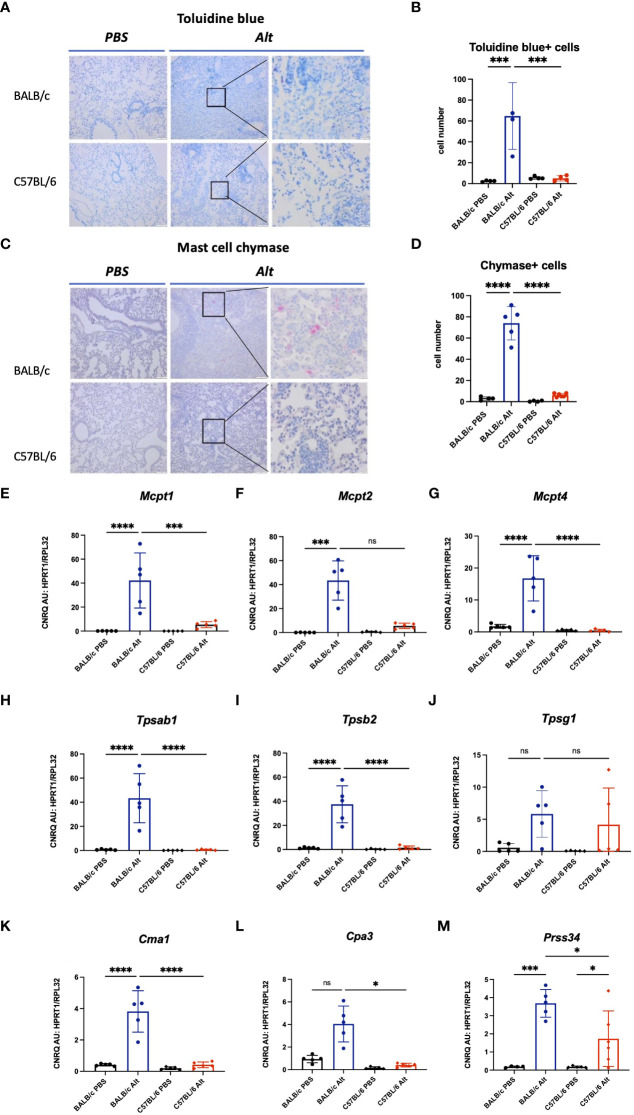
To visualize mast cells, formalin-fixed paraffin-embedded lung sections were stained with toluidine blue **(A)** and the number of mast cells was quantified in four different fields per mouse under magnification of forty times **(B)**. Chymase - positive mast cells in the lungs of mice were visualized by immunohistochemistry **(C)** and quantified **(D)**. Expression of mast cell proteases in the lungs of BALB/c and C57BL/6 mice treated with six intratracheal applications of 20 µg *Alt* extract or PBS. Values of gene expression of *Mcpt1* **(E)**, *Mcpt*2 **(F)**, *Mcpt4* **(G)**, *Tpsab1* **(H)**, *Tpsab2* **(I)**, *Tpsg1* **(J)**, *Cma1* **(K)**, *Cpa3* **(L)** and *Prss34* **(M)** were normalised using reference genes *Hprt1* and *Rpl32*. The data are presented as scatter plot with a bar ± standard deviation (S.D.). Statistical significance between the groups was determined by one-way ANOVA with Sidak’s test for multiple comparisons. n=4-6 per group. *p < 0.05, ***p < 0.001, ****p < 0.0001. ns, not significant.

The lungs of *Alt*-treated BALB/c mice showed increased numbers of mast cells, positive for toluidine blue and chymase compared to PBS controls (**Figures 3A-D**). In agreement with the immunohistochemical staining, the lungs of BALB/c mice treated with *Alt* extract showed increased expression levels of genes encoding mast cell proteases such as *Mcpt1*, *Mcpt*2, *Mcpt4*, *Tpsab1*, *Tpsb2*, *Cma1, Cpa3* (**Figures 3E–L**). As expected, PBS-treated mice lacking significant numbers of mast cells did not express the proteases mentioned above. Only very few mast cells could be found in the lung sections of PBS or *Alt-*treated C57BL/6 mice demonstrating nearly complete lack of mast cell response in C57BL/6 mice treated with *Alt* extract. The gene expression levels of mast cell proteases in the lungs of *Alt*-treated C57BL/6 mice were almost undetectable. The gene expression levels of *Prss34* (*Mcpt11*) were upregulated in both mouse strains with slightly higher levels in *Alt*-treated BALB/c mice than in *Alt*-treated C57BL/6 mice (**Figure 3M**).

### Differential protease expression profiles in bone marrow-derived mast cells of BALB/c versus C57BL/6 mice

**Figure 4:**
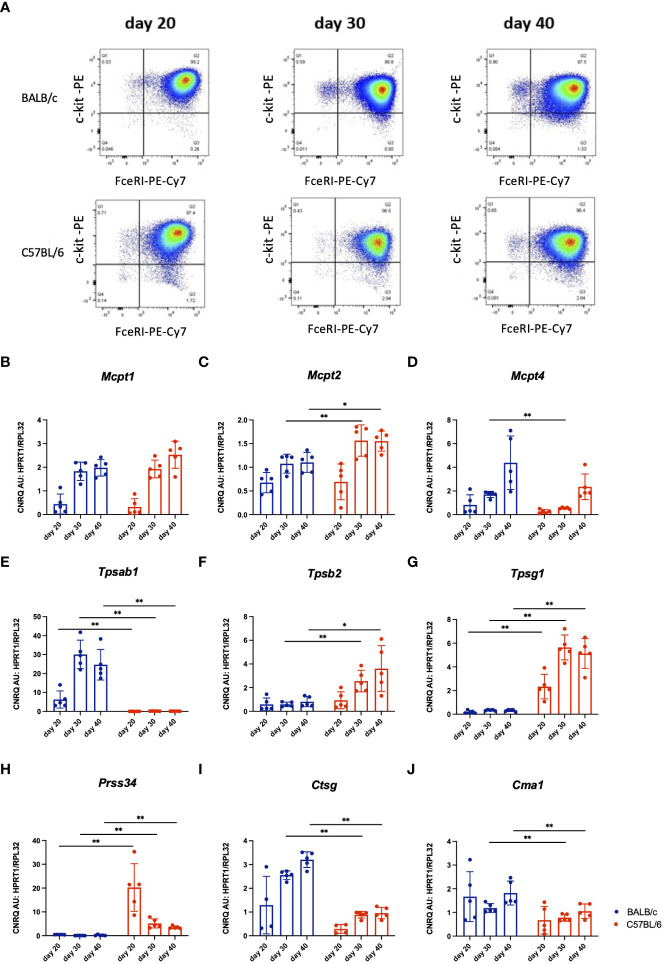
Representative dot plots of *in vitro* differentiated BMMCs from BALB/c and C57BL/6 mice cultured *in vitro* for 20, 30 or 40 days, respectively. Viable mast cells were analyzed for their expression of FceRI and c-kit using flow cytometry **(A)**. Next, gene expression analysis by RT-qPCR of mast cell proteases in these cultured BMMCs from I BALB/c and C57BL/6 mice was performed. Values of gene expression of *Mcpt1* **(B)**, *Mcpt2* **(C)**, *Mcpt4* **(D)**, *TpI1* **(E)**, *Tpsb2* **(F)**, *Tpsg1* **(G)**, *Prss34* **(H)**, *Ctsg* **(I)** and *Cma1* **(J)** were normalised to reference genes *Hprt1* and *Rpl32*. The data represent individual values, bars ± standard deviation (S.D.). Statistical significance between the groups was determined by Mann-Whitney test comparing BALB/c and C57BL/6 mice at each individual time point. The BMMCs were collected from two separate experiments, n=5 per group. *p < 0.05, **p < 0.01.

To test if the enzymatic content of mast cells from C57BL/6 mice might be different from BALB/c mast cells, we have used BMMCs differentiated *in vitro* in the presence of IL-3 as earlier described (24). The purity of BMMCs reached about 97% on day 20 and was consistent from day 20 to day 40 as analysed by expression of c-kit and FcϵRI by flow cytometry (**Figure 4A**). The total RNA was isolated from BMMCs on days 20, 30, and 40 of *in vitro* culture, and the expression levels of several mast cell proteases were tested. RT-qPCR showed a gradual increase from day 20 to day 40 in *Mcpt1, Mcpt2*, and *Mcpt4* gene expression in differentiating BMMCs of both mouse strains (**Figures 4B–D**). Remarkably, while BMMCs from BALB/c mice showed very high expression levels of *Tpsab1* at all differentiation stages tested (**Figure 4E**), the BMMCs from C57BL/6 mice had higher levels of *Tpsb2, Tpsg1*, and *Prss34* (**Figures 4F–H**). The gene expression of *Ctsg* and *Cma1* were significantly lower in BMMCs from C57BL/6 compared to BALB/c mice on days 30 and 40 (**Figures 4I, J**). Thus, despite identical culture conditions, mast cells differentiated *in vitro* from the bone marrow of C57BL/6 and BALB/c mice show a differential protease expression profile.

The difference observed on RT-qPCR between mast cells derived from C57BL/6 versus BALB/c prompted us to perform RNA sequencing (RNAseq) analysis to deeper characterize the phenotype of mast cells derived from different mouse strains. RNAseq analysis was performed in BMMCs of BALB/c and C57BL/6 mice on day 20. In total, 460 genes were upregulated and 263 downregulated in BMMCs of BALB/c mice compared to C57BL/6 mice. A full list of genes is provided in **Supplementary Data** and online repository, accession number GSE216642. 17 genes encoding proteases and 4 protease inhibitors that were differentially expressed between the BMMCs of BALB/c and C57BL/6 are summarized in (**Table 1**). Importantly, the expression of proteases involved in the cleavage of IL-33 is different between the two mouse strains at the baseline, showing that *Cma1* and *Cma2* were upregulated, while *Mcpt4* and *Ctsg* were downregulated in the BMMCs of C57BL/6 mice compared to BALB/c mice.

**Table 1:** List of selected genes differentially expressed by RNAseq analysis in three biological replicates of *in vitro* differentiated mast cells from C57BL/6 as compared to BALB/c.

Genes	Number of reads in C57BL/6 mice	Fold change C57BL/6 compared to BALB/c mice	p - value	p - adj	Protein name	Molecular function
Proteases						
*Cma2*	1610,69	3,58	1,0E-125	1,5E-123	Chymase-2	serine protease
*Fgl2*	18084,38	2,24	2,4E-83	1,9E-81	Fibroleukin	protease
*Gzma*	71,83	7,73	1,1E-18	1,5E-17	Granzyme A	serine protease
*Gzmd*	233,68	8,38	6,2E-69	4,0E-67	Granzyme D	serine protease
*Gzme*	69,27	16,48	7,3E-26	1,4E-24	Granzyme E	serine protease
*Hgfac*	6281,16	2,41	9,9E-114	1,2E-111	Hepatocyte growth factor	serine protease
*Htra1*	1184,94	4,61	1,8E-28	3,7E-27	High-temperature requirement A serine peptidase 1	serine protease
*Cma1/Mcpt8*	4273,77	2,16	2,4E-112	3,0E-110	Chymase-1/Mast cell protease 8	serine protease
*Mcpt9*	1220,34	8,56	2,0E-256	8,9E-254	Mast cell protease 9	serine protease
*Plau*	3686,77	2,00	5,7E-86	4,8E-84	Urokinase-type plasminogen activator	serine protease
*Prcp*	338,50	4,62	2,5E-60	1,4E-58	Lysosomal Pro-X carboxypeptidase	carboxypeptidase
*Prss34*	100,62	48,60	9,2E-33	2,3E-31	Mast cell protease 11	serine protease
*Tpsb2*	99140,01	4,88	3,3E-49	1,4E-47	Tryptase beta-2	serine protease
*Tpsg1*	24719,65	35,37	0,0E+00	0,0E+00	Tryptase gamma	serine protease
*Mcpt1*	5886,78	2,58	4,67E-196	1,38E-193	Mast cell protease 1	serine protease
*Mcpt4*	4770,70	-2,16	2,9E-07	1,7E-06	Mast cell protease 4 chymotrypsin-like activity serine protease	serine protease
*Ctsg*	8137,29	-2,37	1,99E-10	1,60E-09	Cathepsin G	serine protease
protease inhibitors						
*Serpine1*	332,62	9,8	8,33E-86	6,99E-84	Plasminogen activator inhibitor 1	Serine protease inhibitor
*Spink2*	1241,65	-2,3	7,58E-44	2,67E-42	Serine protease inhibitor Kazal-type 2	Serine protease inhibitor
*Serpina3f*	1054,53	-6,39	3,44E-183	8,89E-181	SERPIN domain-containing protein	Serine protease inhibitor
*Serpina3i*	898,69	-3,70	1,15E-89	1,01E-87	Serine (or cysteine) peptidase inhibitor, clade A, member 3I	Serine protease inhibitor

Number of reads, fold difference between the groups, p-value and adjusted p-value (p adj) are presented. The full list of differentially regulated genes is provided in online repository, accession number GSE216642.

### BMMCs from BALB/c mice rapidly degraded IL-33

**Figure 5:**
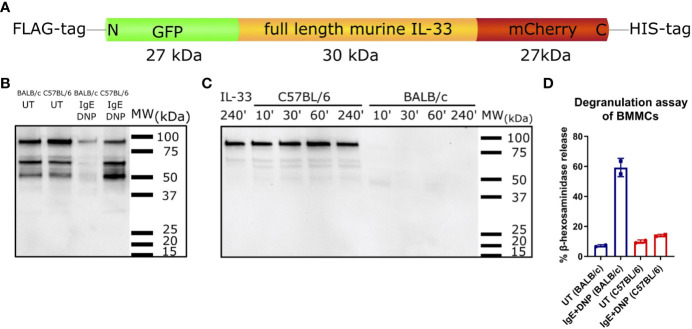
IL-33 degrading abilities of supernatants from C57BL/6 and BALB/c BMMCs. Schematic representation of the GFP-murine IL-33-mCherry fusion protein used for IL-33 cleavage assay with BMMCs supernatants **(A)**. Supernatants from C57BL/6 or BALB/c BMMCs, which were either stimulated with IgE+DNP or left untreated were incubated with GFP-murine IL-33-mCherry for 1 h **(B)**. IL-33 degradation was analysed by western blotting. Supernatants from IgE+DNP stimulated C57BL/6 or BALB/c BMMCs were incubated with GFP-murine IL-33-mCherry for 10, 30, 60, or 240 min at 37°C. IL-33 incubated only in buffer for 240 min at 37°C served as negative control. IL-33 degradation was analysed by western blotting **(C)**. Beta-hexosaminidase degranulation assay of untreated or IgE+DNP stimulated BMMCs from C57BL/6J or BALB/c mice **(D)**. Data are presented as mean ± SEM.

Next, we tested whether the full-length IL-33 protein could be processed *in vitro* by mast cell supernatants of BMMCs of both mouse strains. GFP-murine IL-33-mCherry fusion protein (**Figure 5A**) was used for a cleavage assay with BMMCs supernatants. DNP-IgE/DNP activated BMMCs of C57BL/6 mice did not degrade IL-33, while supernatants of similarly activated BALB/c BMMCs effectively degraded IL-33 after 10 min of co-incubation (**Figures 5B, C**). Importantly, the efficiency of IL-33 cleavage was linked to the degree of mast cell degranulation after IgE crosslinking. C57BL/6 BMMCs showed a much weaker release of β-hexosaminidase in response to IgE-DNP complex formation than BALB/c BMMCs (**Figure 5D**).

## Discussion

It is well-known that mast cells play a central role in mediating allergic diseases. Mast cells contribute to the activation of immune cells through their broad range of mediators. Next to proteases and histamine, they can also release cytokines. It is known that TNF-α, IL-4, and IL-13 release is triggered by IL-33 and drives the type 2 inflammatory pattern seen in asthma (25–29). We have previously shown that the inflammatory response to *S. aureus* protease like protein D (SplD) and *Alt* extract, which are IL-33 dependent asthma models, can vary among different genetic backgrounds in mice. In these models, a stronger eosinophilic response was found in C57BL/6 than BALB/c mice (12, 30, 31). Similar observations were made in other type 2 response inducing asthma models of ovalbumin-alum and house dust mite (32–35). We, therefore, hypothesized in this work that mast cells might differ between C57BL/6 and BALB/c mice, leading to a different inflammatory response.

In this study, we provide a comprehensive comparative analysis of BMMCs from BALB/c and C57BL/6 mice showing significant differences in expression of major mast cell proteases and protease inhibitors. RNA sequencing analysis of BMMCs *in vitro* from both strains has shown that tryptase genes were differentially expressed, namely C57BL/6 mice lacked *Tpsab1* expression but had higher levels of *Tpsb2* and *Tpsg1* instead, while *Ctsg* and *Cma1* were higher in BMMCs from BALB/c mice. It is known that mast cell chymase and cathepsin G can also generate more potent forms of IL-33 through its enzymatic processing (9). Some genetic differences were reported earlier, such as mutations in mMCP-7 of C57BL/6 mice that leads to its loss of expression (36). A frameshift mutation in the protease inhibitor *Serpina3i* that leads to a truncated and probably unfunctional protein in BALB/c mice (31). Their role in the differential regulation of IL-33 has been suggested (30).

Moreover, we here demonstrate *in vitro* that activated BMMCs from C57BL/6 and BALB/c mice show different enzymatic activity towards IL-33. Mast cells are known to regulate the processing of IL-33 in a dichotomic way: their proteases degrade IL-33 (37, 38) or generate active processed forms of IL-33 *in vitro* (9), generating a more functionally potent form of IL-33. The observed functional differences between mast cells of C57BL/6 and BALB/c mice could be explained by their different protease content and their IgE-dependent activation potential. A lower degranulation activity by IgE cross linkage of C57BL/6 bone marrow mast cells than BALB/c mast cells has also recently been demonstrated elsewhere (39). However, the exact mechanisms leading to this immune phenotypic difference need to be addressed in future studies. Mast cells of rodents are classically distinguished into two different phenotypes, mucosal and connective tissue mast cells. Connective tissue mast cells express high levels of chymases (Mcpt-4, Mcpt-5) and tryptases (Mcpt-6, Mcpt-7), while mucosal tissue mast cells mostly show an upregulation of Mcpt-1 and Mcpt-2 (40). The transcription program of mast cells varies depending on their differentiation stage (41, 42) and organ-specific localization (43). The studies were mostly performed in C57BL/6 mice, while the information on BALB/c mast cell expression profile is scarce (41, 42). In our experiments, we have seen an upregulation of markers characteristic for both types of mast cells in the lungs of BALB/c mice receiving *Alt* extract with a strong increase of *Mcpt-1, Mcpt-2, Mcpt-4, Mcpt-6* and *Mcpt-7* next to an upregulation of *Cma1* and *Cpa3*. In C57BL/6 mice, a nearly complete absence of mast cells and their proteases was seen in the lungs after the treatment with *Alt*. Even though *Prss34* expression was increased in *Alt*-treated C57BL/6 mice, BALB/c mice treated with the *Alt* extract had even higher levels of *Prss34*.

In our model, we repeatedly exposed mice to *Alt* extract as an *in vivo* mouse model of allergic IL-33-dependent airway inflammation, because *Alt* triggers the airway epithelium resulting in the release of alarmins, including ATP, IL-33, and TSLP, which induce ILC2 proliferation and a type 2-driven immune response (4, 44). In our study, BALB/c mice respond to *Alt* with features of allergic airway inflammation, including strong eosinophilic inflammation and neutrophilia in the BALF and lungs, in agreement with previous studies (4, 12, 44). Further, as seen previously, C57BL/6 mice presented an even higher eosinophilic response in the BALF as well as higher lung IL-5 levels than BALB/c mice. Based on the low numbers of mast cells and the low mast cell protease expression levels in the lungs of C57BL/6 mice compared to BALB/c mice, we suggest a protective effect of mast cells, that dampen IL-5 and the eosinophilic response in BALB/c mice.

Interestingly, IL-33 protein levels were remarkably higher in the lungs of BALB/c mice treated with *Alt*. We have noted before that IL-33 protein levels by Luminex, however, do not reflect the levels of cleaved IL-33 (IL-33_C_) found by western blotting (12). We demonstrated different processing patterns of IL-33 seen by western blotting between BALB/c mice with the prevailing IL-33_C_ and C57BL/6 mice, where also full-length IL-33 was detected. Therefore, we hypothesize that this different cleavage pattern is a consequence of different proteolytic regulation of IL-33 by endogenous proteases upon intratracheal treatment with the *Alt* extract. Based on our previous results, we expect that Mcpt-4 present in the lungs of BALB/c mice degrades IL-33 (31). Next to mast cell proteases, also neutrophilic proteases, such as proteinase 3, and caspases can degrade IL-33 and inactivate it (10, 45). We recently demonstrated the importance of neutrophil proteases in the regulation of IL-33 in a comparable *Alt*-induced asthma model (12). Consistent with our previous results, we here showed that BALB/c mice respond with a stronger neutrophilic inflammation than C57BL/6 mice, which, in addition to the mast cell proteases, influences the IL-33 signaling pathway. In our previous study, depletion of neutrophils *in vivo* by *i.p.* injections of anti-Ly6G antibodies in *Alt*-treated BALB/c mice resulted in a significant decrease of elastase and reduced processing of IL-33_C_ supporting the additional effect of neutrophilic proteases in the regulation of IL-33 processing (12). However, in our previously used SplD-induced asthma model, where C57BL/6 mice also presented a stronger eosinophilic response than BALB/c mice, the neutrophilic response was negligibly weak, and still IL-33 was processed in a comparable way to the here shown *Alt*-induced allergic airway inflammation model, which underlines the importance of mast cell proteases in the regulation of IL-33 (22). Remarkably, the expression levels of ST2 receptor were comparable between the lungs of *Alt* -treated C57BL/6 and BALB/c mice, excluding the role of possible deficient IL-33/ST2 signaling axis in this regulation.

To conclude, our study demonstrates that mast cells differ in number and protease content between the two mouse strains most often used in asthma and allergy research. These differences might influence the phenotypic outcome in IL-33-dependent models of type 2 inflammation which are unlikely to be related to different efficiency of the IL-33/ST2 signalling axis. The mast cells and their proteases play a regulatory role in IL-33-induced inflammation by limiting its proinflammatory effect *via* the IL-33 signalling pathway because the models of allergic inflammation tested including BALB/c mice show a weaker eosinophilic response compared to C57BL/6 mice.

## Materials and methods

### Mice: experimental procedures

All experimental procedures were approved by the local Ethical Committee of Ghent University. Seven-week-old female BALB/c or C57BL/6J wild-type mice (Janvier, Saint-Berthevin, France) received six intratracheal applications of 50 µl PBS alone or 20 µg of *Alt* extract (Stallergenes Greer, London, UK) in 50 µl PBS. Animals were kept in individually ventilated cages in a 12-hour/12-hour light/dark cycle. The experiments were performed under light gaseous anaesthesia with isoflurane/air (Ecuphar, Breda, The Netherlands). Mice were euthanized with an intraperitoneal (i.p.) injection of 150 µl Dolethal (Vétoquinol, Lure, France). Lungs were perfused with 0.9% NaCl. For the cytokine analysis, a piece of the lung was snap-frozen in liquid nitrogen and stored at -80°C for further analysis. To perform immunohistochemical staining a piece of the lung was immersed in 10% formalin and paraffin-embedded. The 5μm sections were cut and used for the subsequent staining.

### Flow cytometry

BALFs were collected with PBS containing protease inhibitor cocktail (Roche, Mannheim, Germany), 0.5% bovine serum albumin and ethylenediaminetetraacetic acid (EDTA) (Sigma-Aldrich, Bornem, Belgium). The lungs were enzymatically dissociated by incubation in 1 mg/ml collagenase type II (Worthington Biochemical, New Jersey, USA) at 37°C for one hour shaking to obtain single cell suspension. Red blood cells were lysed by cell lysis solution (VersaLyse Lysing solution, Beckman Coulter, Marseille, France). 1 x 10^6^ cells were stained with the following antibodies: purified CD16/CD32 (clone 93), CD4-FITC (clone RM4-4), CD8a-PerCp-Cy5.5 (clone 53-6.7), CD11c-PE-Cy7 (clone HL3), CD11b-PerCP-Cy5.5 (clone M1/70) and Gr1-FITC (clone RB6-8C5) purchased from Thermo Fisher Scientific and Siglec F-PE (clone ES22-10D8) from Miltenyi Biotec (Bergisch Gladbach, Germany). To exclude the dead cells the LIVE/DEAD Fixable Near-IR Dead Cell Stain Kit (Merck Millipore, Massachusetts, USA) was used. The following gating strategy was applied for flow cytometry to analyze eosinophils and neutrophils in BALF and lungs. First, the subset of viable cells was gated through the selection of singlets viable cells. Next from CD11c^-^ population eosinophils were defined as CD11b^+^, SiglecF^+^, and neutrophils as SSC^low^, CD11b^+^, Gr1^+^ as earlier described in (12). CD4^+^ and CD8^+^ T cells were gated as subsets of singlet/viable SSC^low^ cells. Murine lungs and BALF were analysed by flow cytometry using the FACS Canto II (BD Biosciences, Erembodegem, Belgium).

### Luminex assays

A Luminex analysis was performed to measure the protein concentration of IL-4, IL-5, IL-13, IL-25, IL-33, and G-CSF in mouse lung homogenates. The mouse magnetic Luminex kit (R&D Systems, Minnesota, USA) was used and all steps were performed according to the manufacturer’s protocol. The lung homogenates (1 mg/ml) were diluted 1/2 by adding 25 μl of each sample to 25 μl Calibrator Diluent RD6-52. A standard curve for each cytokine analysed was generated using a series of dilution (diluted 1/3) of the Standard Cocktails in Calibrator Diluent RD6-52. A diluted biotin-antibody cocktail and streptavidin- phycoerythrin (PE) solution were made according to the manufacturer’s protocol in a 1/10 dilution. The plate was read using the Bio-Rad analyzer, Bioplex 200 system with Luminex xMAP technology (Bio-Rad, California, USA).

### Total IgE analysis

The levels of total IgE in serum of mice were analyzed with an ELISA kit from ThermoFisher Scientific according to manufacturer instructions. The reaction was read using automated spectrophotometric plate reader and the SkanIt Software (Multiscan FC, Thermo Fisher Scientific).

### *In vitro* differentiation of murine bone marrow-derived mast cells

The mast cells were differentiated *in vitro* from BMMCs of naive wild type BALB/c and C57BL/6 mice. The BMMCs were differentiated for 40 days in Dulbecco’s modified Eagle’s medium with 10% fetal bovine serum, L-glutamine, penicillin/streptomycin and 1 mM sodium pyruvate (all from Thermo Fisher Scientific) supplemented with 5 ng/ml IL-3, 5 ng/ml IL-9, 1 ng/ml TGF-β1 and 30 ng/ml stem cell factor (all from PeproTech, London, UK). On days 20, 30 and 40 of the culture, the purity of obtained mast cells was tested by flow cytometry using anti-c-Kit-PE and anti-FcϵRI-PE-Cy7 antibodies (Thermo Fisher Scientific). The representative dot plots are provided in **Figure 4A**. The experiment was repeated with four separate cultures. RNA from murine bone marrow-derived mast cells was isolated on days 20, 30 and 40.

### BMMCs stimulation

BMMCs were stimulated by DNP-IgE complex formation. 2x10^7^cells/mL of BMMCs were preincubated with 0.15 µg/mL anti-DNP IgE (Sigma-Aldrich) overnight and stimulated for 1 h with 200 ng/mL DNP-HSA (Sigma-Aldrich) in Tyrodes buffer (10 mM HEPES, 130 mM NaCl, 6.2 mM D-glucose, 3.0 mM KCl, 1.4 mM CaCl_2,_ 1.0 mM MgCl_2_ and 0.1% BSA, pH 7.15, sterile filtered) at 37°C. Supernatants were frozen and stored at -20°C until usage.

### IL-33 cleavage assay

Supernatants were incubated with 1µg of a full-length murine IL-33 GFP and mCherry fusion protein in a total volume of 20µL for 10, 30, 60 or 240 min. IL-33 degradation was detected by Western blot using mouse IL-33 antigen affinity-purified polyclonal goat IgG (dilution 1:300; R&D Systems) with polyclonal peroxidase labelled anti-goat IgG (H+L) (1:1000; Vector Laboratories Inc., Burlingame, CA, USA).

### Beta-hexosaminidase degranulation assay

Degranulation of BMMCs from C57BL/6 and BALB/c mice was tested as previously described (22).

### Immunohistochemistry

Formalin-fixed paraffin-embedded lung sections were deparaffinized and dehydrated. The slides were exposed to citrate buffer for antigen retrieval and endogenous peroxidase was blocked by incubation in 3% H_2_O_2_. Lung sections were stained with anti-mast cell chymase (Abcam) and anti- IL-33 (R&D systems). The ImmPRESS^®^-AP Horse Anti-Goat IgG Polymer Detection Kit, Alkaline Phosphatase (Vectorlabs, Burlington, California, USA) and Dako REALTM Detection System, Alkaline Phosphatase/RED, Rabbit/Mouse (Agilent Technologies, Santa-Clara, California, USA) was used to visualize the binding. The slides were counterstained with haematoxylin and mounted with Aquatex (Sigma-Aldrich). The cells positive for IL-33 or chymase were counted in five power fields per mouse lung at magnification of forty times.

### Toluidine blue staining

Formalin-fixed paraffin-embedded murine lung sections were deparaffinized and dehydrated and stained for 15 minutes with toluidine blue (Sigma-Aldrich) working solution at pH 2.3. The slides were air-dried and mounted with a non-aqueous medium (Pertex, 00811, Histolab). The images were taken using Nikon Eclipse Ni-U upright microscope with NIS BR imaging software.

### Reverse transcription quantitative polymerase chain reaction

Snap frozen mouse tissue samples (± 20-25 mg) were disrupt using a mortar and pestle containing liquid nitrogen and thawed directly into lysis solution (QIAGEN, Antwerp, Belgium), while murine bone marrow-derived mast cells were directly vortexed for 5 min in lysis solution. The lysate was then homogenized using a QIAshredder homogenizer (QIAGEN). The RNA isolation was further performed using the RNeasy Mini Kit (QIAGEN) following the manufacturer’s instructions. The RNA concentration was measured with Nanodrop (Thermo Fisher Scientific, Waltham, Massachusetts, United States) and the quality of the RNA was assessed with the Fragment Analyzer™ Automated CE System (Agilent technologies, California, USA). 500 ng of the isolated RNA was transcribed into cDNA with the iScript Advanced cDNA Synthesis Kit (Bio-Rad) and subsequently diluted with nuclease-free water to 2.5 ng/µl cDNA (total RNA equivalent). Real-time PCR amplifications were performed in a 384-well plate LightCycler LC480 System (Roche Diagnostics, Mannheim, Germany). qPCR reactions (5 µl) contain 5 ng cDNA, 2x SsoAdvanced Universal SYBR Green Supermix (Bio-Rad) and 250 nM forward and reverse primer (**Table 2**). The PCR protocol consisted of 2 minutes polymerase activation at 95°C and 44 cycles of 5 seconds at 95°C, 30 seconds at 60°C and 1 second at 72°C followed by a dissociation curve analysis from 60°C to 95°C. For murine BMMCs, RT-qPCR was performed for mouse mast cell proteases (*Mcpt*1, Mcpt2, Mcpt4), tryptase α/β1 (*Tpsab1*), tryptase β2 (*Tpsb2*), tryptase γ1 (*Tpsg1*), serine protease 34 (*Prss34*), cathepsin G (*Ctsg*) and mast cell chymase 1 (*Cma1*). For mouse lung tissue, RT-qPCR was performed for mouse mast cell proteases (*Mcpt*1, Mcpt2, Mcpt4), tryptase α/β1 (*Tpsab1*), tryptase β2 (*Tpsb2*), tryptase γ1 (*Tpsg1*), mast cell chymase 1 (*Cma1*), carboxypeptidase A3 (*Cpa3*) and serine protease 34 (*Prss34*). The primer sequences of all target genes were shown in **Table 2**. *IL1rl1* (encoding ST2) Taqman (QuantiTect Probe PCR kit; Mm00516117_ml) was purchased from Applied Biosystems. The expression of 2 reference genes, hypoxanthine phosphoribosyltransferase 1 (*Hprt1*) and ribosomal protein L32 (*Rpl32*) was used to normalize for transcription and amplification variations among samples after a validation with geNorm (Biogazelle, Zwijnaarde, Belgium). The normalized relative quantities (NRQs) were calculated with the qBase^+^ software (Biogazelle, Belgium) and the final gene expression levels are expressed as NRQs per 5 ng cDNA.

**Table 2:** List of primers used in the study.

Gene name	Primer sequence FW (5’-3’)	Primer sequence RW (5’-3’)
*Hprt1*	CCTAAGATGAGCGCAAGTTGAA	CCACAGGACTAGAACACCTGCTAA
*Rpl32*	GGCACCAGTCAGACCGATATG	CCTTCTCCGCACCCTGTTG
*Mcpt1*	AAAAACAGCATACATGGGAG	CATATGCAGAGATTCTGGTG
*Mcpt2*	CAATAGGACAAGGAGATTCTG	TAATAGGAGATTCGGGTGAAG
*Mcpt4*	CACTGTAGTGGAAGAGAAATC	GAGGAATTACATTCACAGAGG
*Tpsab1*	AAACCCTGTGAACATTTCTG	TACACCATTGTCGATGTTAC
*Tpsb2*	GACATTGATAATGACGAGCC	GACAATGGGAAAATCATCTCC
*Tpsg1*	TTCTCTGGGTCTGTGAAC	GTTTTACAGTGGAGAAGTGG
*Prss34*	AGTCTATGGTGTCCCTTAAC	ATGGTAAGGAGGGAATATGG
*Ctsg*	TGACCTTTATTCTACTCCAAGG	GTAACATTTATGGAGCTTCCC
*Cma1*	GTATACAAGGGAGACTCTGG	CAGAGTTAATTCTCCCTCAAG
*Cpa3*	ATGGCTACACATTCAAACTG	TATTGGGCCGTAGATGTAAC

### Western blotting

Tissue homogenates of 20 - 25 mg of mouse lung tissue were made by a mechanical dissociation in a mixture of tissue lysis buffer (Thermo Fisher Scientific) and protease inhibitor cocktail (Roche, Sigma-Aldrich, Merck KGaA, Darmstadt, Germany) with a TissueLyser LT (QIAGEN). The supernatant was collected and centrifuged for 10 min at 3000 rpm at 4°C. The tissue homogenate was stored in -20°C. For spectrophotometric determination of the concentration of total proteins in the tissue homogenate, a standard series of dilution with bovine serum albumin (Sigma- Aldrich, Merck KGaA, Darmstadt, Germany) was used. Homogenate samples were diluted 1:20 with NaCl (0,9%) (Versylene Fresenius). The dye solution was prepared by diluting 1 part protein assay dye reagent concentrate (Bio-Rad) with 4 parts distilled water. Subsequently, the optical density was measured at a wavelength of 570 nm using the SkanIt Software (Multiscan FC, Thermo Fisher Scientific). Samples of mouse lung homogenates were prepared for gel electrophoresis with 25 μg protein. For gel electrophoresis, Mini-protean TGX stain-free precast gels (Bio-Rad) were used and 10 μl of the Precision Plus Protein Dual Color Standards (Bio-Rad) was added. The proteins were blotted on a nitrocellulose membrane (Bio-Rad). After blotting, the membrane was washed with a washing buffer and adding a blocking buffer (5% skim milk powder in 1x TBST), the membrane was incubated with the primary antibody (goat anti-IL-33, 0,6 μg/ml)) overnight at 4°C while shaking. After washing, the membrane was incubated with the appropriate secondary antibody (anti-goat IgG horseradish peroxidase (HRP) (1 μg/ml)). Visualization was done with the Chemidoc imaging system (Bio-Rad) after incubation with SuperSignal West Dura (Thermo Fisher Scientific).

### RNAseq analysis, RNAseq pipeline and data quantification

RNA was isolated from 1*10^6^ murine mast cells per condition as described above. The integrity of RNA was tested using Fragment Analyzer and 100 ng of total RNA was used for RNA sequencing analysis. The TruSeq Stranded mRNA kit (Illumina) was used to prepare a RNAseq library according to the manufacturer’s protocol, followed by PE100 cycles sequencing on one lane of a NovaSeq 6000 S1 run (Illumina).

### Bioinformatics

All fastq files passed quality control with FastQC v0.11.5 (46). Salmon v0.8.2 (47) was used to align raw RNAseq reads against Ensembl *Mus musculus* GRCm39 and get quantification estimates at the transcript level. All subsequent analyses were conducted in R software v 4.0.3 (48). Differential analysis was computed using tximport v1.10.0 (49) and the negative binomial generalized linear modeling implemented in DESeq2 package version 1.30.1 (50) Genes were regarded to be differentially expressed when the q value cutoff (FDR adjusted p-value using Benjamini–Hochberg mode l (51) was lower than 0.05. In order to obtain significantly different genes, we set the selection criteria as: the multiple of difference| Fold Change| > 2. To visualize the differentially - expressed (DE) genes identified using the methods described above, the Bioconductor Enhanced Volcano (52) and g plots (53) packages were used. Enhanced Volcano was used to display each gene’s shrunken log2 fold change (LFC) against its adjusted p-value. A gene classification and functional annotation analysis were performed using the ‘Gene Functional Classification’ and ‘Functional Annotation Charts’ tools of the Database for Annotation, Visualization and Integrated Discovery (DAVID Bioinformatics Resources 6.8, NIAID/NIH) (54). As annotation category, KEGG_PATHWAYS was selected for pathways analysis.

### Statistical analysis

The data were analysed using D’Agostino & Pearson test for normal (Gaussian) distribution (alpha = 0.05). When three or more experimental groups were compared, the normally distributed data were analysed by parametric one-way ANOVA with Dunnett’s multiple comparisons test. The data, which were not normally distributed, were analysed by non-parametric Kruskal-Wallis test with Dunn’s test for multiple comparisons. Significance was determined as followed: * p<0.05, ** p<0.01, *** p<0.001, **** p<0.0001.

## Data availability statement

The data presented in the study are deposited in the GEO repository, accession number GSE216642.

## Ethics statement

The animal study was reviewed and approved by Ethical Committee of Ghent University, Faculty of Medicine and Health Sciences.

## Author contributions

OK designed and performed experiments, analyzed data and wrote the original draft. DK and MI performed the bioinformatics analysis of the RNA sequencing dataset. AT performed experiments and revised the manuscript. JV, JS and AD collected experimental material, did immunohistochemistry and western blotting, analyzed data, revised the manuscript. SV contributed to the collection of the murine samples and revised the manuscript. GH performed and analyzed Luminex data. ND performed and analyzed RT-qPCR data. FC performed RNA seq analysis. HB provided a genetic construct encoding full length IL-33. MV, and DVK supervised the study, provided analysis tools, revised the manuscript. CB supervised the study, provided analysis tools, revised the manuscript. All authors discussed the results and approved the manuscript. All authors contributed to the article and approved the submitted version.

## Supplementary material

Supplementary Figure 1 The levels of IL-4 **(A)**, G-CSF **(B)**, and IL-25 **(C)** in the lung homogenates were measured by Luminex. The data are presented as a scatter plot with a bar ± standard deviation (S.D). Statistical analysis was performed by one-way ANOVA with Dunn’s test for multiple comparisons. N=4-6 per group.

Supplementary Table 1 List of genes differentially expressed in RNA-Seq analysis of *in vitro* differentiated mast cells (BMMCs) from naïve BALB/c and C57BL/6 mice obtained from three biological replicates.
