# Supplementary figures and images for: Differential protease content of mast cells and the processing of IL-33 in Alternaria alternata induced allergic airway inflammation in mice
Source: Front Immunol. 2023 Apr 19;14:1040493. doi: 10.3389/fimmu.2023.1040493 (PMC10154570; doi:10.3389/fimmu.2023.1040493)

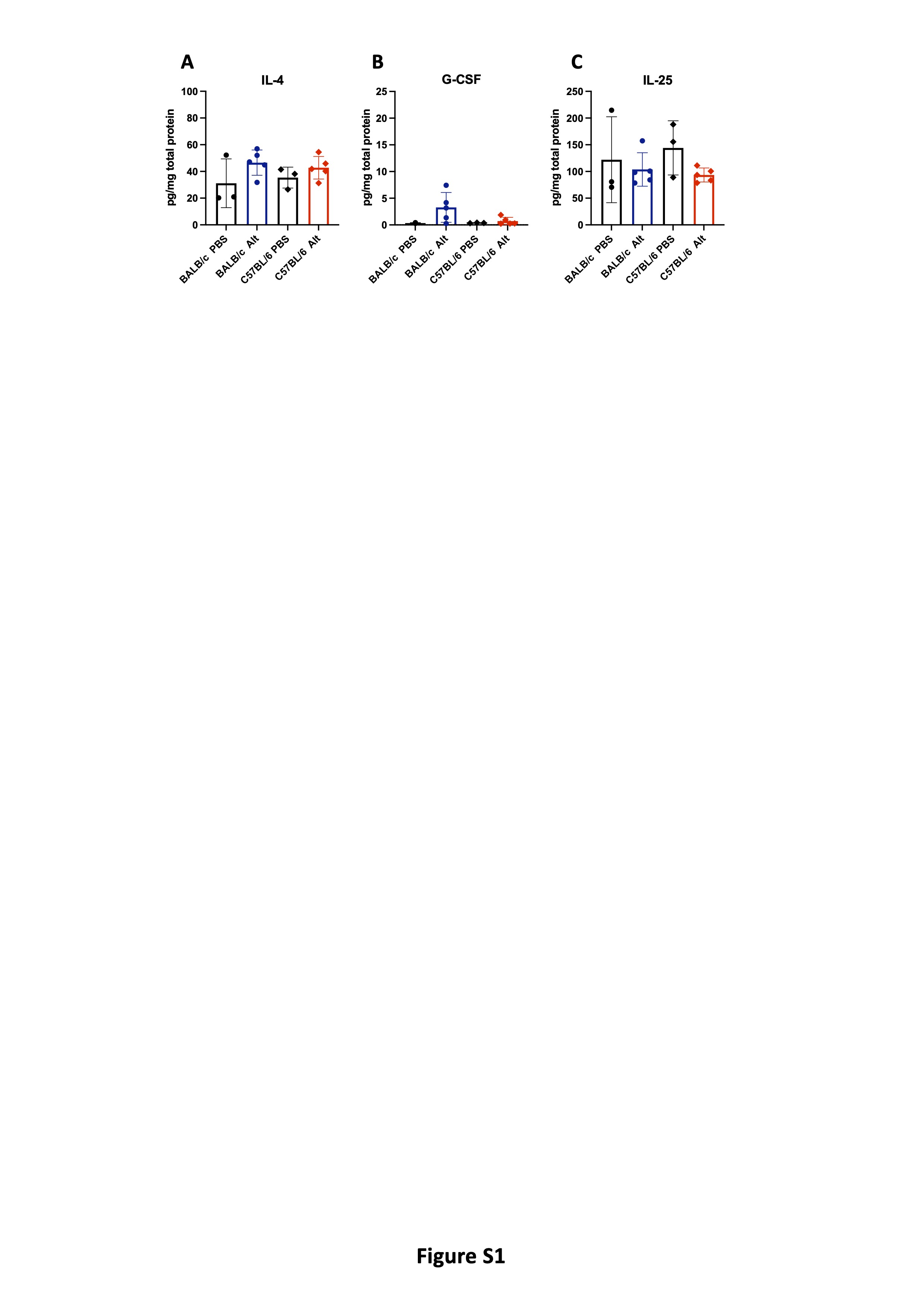

Supplement: Supplementary Figure 1 — The levels of IL-4 (A), G-CSF (B), and IL-25 (C) in the lung homogenates were measured by Luminex. The data are presented as a scatter plot with a bar ± standard deviation (S.D). Statistical analysis was performed by one-way ANOVA with Dunn’s test for multiple comparisons. N=4-6 per group. [file Image_1.jpg]
